# Mitochondrial GRIM-19 as a potential therapeutic target for STAT3-dependent carcinogenesis of gastric cancer

**DOI:** 10.18632/oncotarget.9167

**Published:** 2016-05-04

**Authors:** Yi Huang, Meihua Yang, Huajian Hu, Xiaodong Zhao, Liming Bao, Daochao Huang, Lihua Song, Yang Li

**Affiliations:** ^1^ Chongqing Key Laboratory of Child Infection and Immunity, Children's Hospital of Chongqing Medical University, Ministry of Education Key Laboratory of Child Development and Disorders, China International Science and Technology Cooperation Base of Child Development and Critical Disorders, Chongqing 400014, PR China; ^2^ Department of Neurosurgery, Xinqiao Hospital of Third Military Medical University, Chongqing 400038, PR China; ^3^ Department of Gastroenterology, Children's Hospital of Chongqing Medical University, Chongqing 400014, PR China; ^4^ Department of Pathology, Dartmouth-Hitchcock Medical Center, Geisel School of Medicine at Dartmouth College, Lebanon, NH 03756, USA; ^5^ Animal Care Center, Children's Hospital of Chongqing Medical University, Chongqing 400014, PR China; ^6^ Department of Gastroenterology, 416 Hospital of Nuclear Industry, Chengdu 610051, PR China

**Keywords:** GRIM-19, gastric cancer, chronic atrophic gastritis, clinical outcome, STAT3

## Abstract

Aberrant STAT3 activation occurs in most human gastric cancers (GCs) and contributes to the malignant progression of GC, but mechanism(s) underlying aberrant STAT3 remain largely unknown. Here we demonstrated that the gene associated with retinoid interferon-induced mortality 19 (GRIM-19) was severely depressed or lost in GC and chronic atrophic gastritis (CAG) tissues and its loss contributed to GC tumorigenesis partly by activating STAT3 signaling. In primary human GC tissues, GRIM-19 was frequently depressed or lost and this loss correlated with advanced clinical stage, lymph node metastasis, *H. pylori* infection and poor overall survival of GC patients. In CAG tissues, GRIM-19 was progressively decreased along with its malignant transformation. Functionally, we indentified an oncogenic role of GRIM-19 loss in promoting GC tumorigenesis. Ectopic GRIM-19 expression suppressed GC tumor formation *in vitro* and *in vivo* by inducing cell cycle arrest and apoptosis. Moreover, we revealed that GRIM-19 inhibited STAT3 transcriptional activation and its downstream targets by reducing STAT3 nuclear distribution. Conversely, knockdown of GRIM-19 induced aberrant STAT3 activation and accelerated GC cell growth *in vitro* and *in vivo*, and this could be partly attenuated by the blockage of STAT3 activation. In addition, we observed subcellular redistributions of GRIM-19 characterized by peri-nuclear aggregates, non-mitochondria cytoplasmic distribution and nuclear invasion, which should be responsible for reduced STAT3 nuclear distribution. Our studies suggest that mitochondrial GRIM-19 could not only serve as an valuable prognostic biomarker for GC development, but also as a potential therapeutic target for STAT3-dependent carcinogenesis of GC.

## INTRODUCTION

Gastric cancer (GC) is the second leading cause of cancer-related death worldwide [1, 2]. Despite accelerated progress in diagnosis and treatments, the biological and molecular mechanisms underlying GC development are still incompletely understood. The aggressive nature of GC is related to mutations of various oncogenes and tumor suppressor genes and their aberrant downstream signal transduction pathways involved in the control of many aspects of cancer biology [3–5]. Signal transducer and activator of transcription 3 (STAT3) has been receiving considerable attention for its oncogenic role in carcinogenesis and malignant transformation of inflammation-associated diseases [6–11]. Aberrant STAT3 occurs in most GCs and contributes to oncogenesis and aggressive GC biology by the upregulation of critical genes [6–8, 12–14] and the dysregulation of cell growth and survival [1–2, 5–8, 13–14]. Therefore, STAT3 was identified as a novel prognosis biomarker in GC patients and could serve as a potential therapeutic target for human GC [6–7, 15–17]. However, at present, mechanism(s) underlying aberrant STAT3 activation in GC patients remain largely unknown.

The combination of IFN-β and all-trans retinoic acid (RA) has been shown to synergistically inhibit tumor activity in a number of animal models and in clinical studies [18–19]. Gene associated with retinoid-IFN-induced mortality 19 (GRIM-19), one of the IFN/RA-inducible GRIM products [20], was originally identified as a potential tumor suppressor associated with growth inhibition or cell apoptosis [20–23]. Subsequently, GRIM-19 was identified as an essential subunit of the mitochondrial respiratory chain (MRC) complex I [22–25] and was reported to repress STAT3 activation via functional binding [26–29]. More recently, altered expression or mutation of GRIM-19 has been further uncovered in numerous human cancers [30–34]. Its functional role in growth inhibition or cell apoptosis seems to be cell type-dependent [20, 22]. Our previous work has demonstrated a suppressive aspect of GRIM-19 in the GC metastasis [21]. However, the functional role and clinical relevance of GRIM-19 in gastric carcinogenesis remain poorly understood. Notably, the potential mechanism of GRIM-19 to inhibit STAT3 has been investigated [24–27], but the exact cellular localization of GRIM-19, which is critical to its interaction with STAT3 [27], is still controversial and is yet to be determined [20, 24, 27]. Therefore, systematic investigations are needed to clarify the correlation of GRIM-19 expression with aberrant STAT3 activation in GC cells.

To this end, we investigated the functional role of GRIM-19 expression and its clinical implications in the pathogenesis of GC, as well as precancerous chronic atrophic gastritis (CAG). We observed that a frequent downregulation or loss of GRIM-19 in primary human GC tissues and this loss is associated with aggressive clinicopathologic features of patients with GC. In CAG tissues, GRIM-19 was also severely depressed or lost with progressive decreases in parallel with malignant transformation of CAG. Through gain- and loss-of-function strategies, we indentified an oncogenic role of GRIM-19 loss in promoting GC tumorigenesis partly by activating a STAT3-dependent pathway. Ectopic expression of GRIM-19 in human GC cells inhibited STAT3 activation and its downstream targets to induce cell cycle arrest and cell apoptosis *in vitro* and to suppress tumor formation *in vivo*. Conversely, knockdown of GRIM-19 accelerated GC cell growth *in vitro* and *in vivo*, and this was attenuated by the blockage of STAT3 activation. At the subcellular level, GRIM-19 expression exhibited subcellular redistributions characterized by peri-nuclear aggregates, cytoplasmic non-mitochondria distribution and nuclear invasion, which should be responsible for reduced STAT3 nuclear distribution. These studies collectively suggest that GRIM-19 may function as not only a potential prognostic marker of human GC, but also a prospectively therapeutic target for GC treatment.

## RESULTS

### GRIM-19 expression is severely depressed or lost in human GC

**Figure 1:**
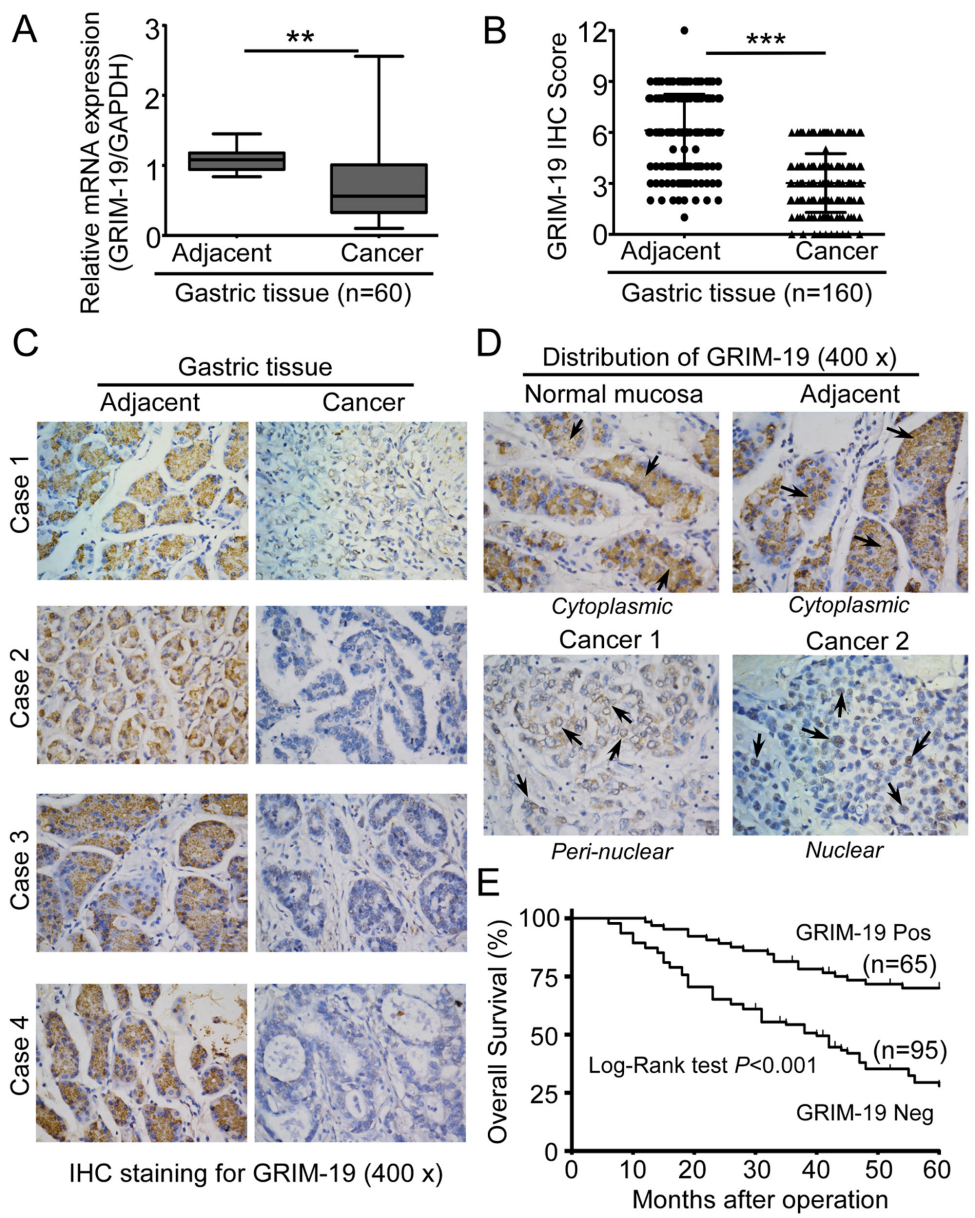
GRIM-19 is severely depressed or lost in human GC tissues **A.** Reduced GRIM-19 mRNA levels in GC tissues. Total RNA was extracted from paraffin- embedded human primary GC tissues and paired adjacent tissues (n=60). GRIM-19 mRNA was detected by qRT-PCR. GAPDH was used as an internal control. **B.** Decreased expression of GRIM-19 protein in GC tissues. GRIM-19 protein levels were detected by specific GRIM-19 antibody in primary GC tissues and corresponding adjacent tissues (n=160) using IHC staining. GRIM-19 IHC scores were analyzed on the basis of the intensity of GRIM-19 staining and the percentage of GRIM-19-positive tumor cells as described in Materials & Methods. Representative images of GRIM-19 staining in GC tissues and adjacent tissues are shown **C. D.** Cell distribution of GRIM-19 in normal gastric mucosa, adjacent and GC tissues. GRIM-19 distribution in these tissues was indicated by black arrows. Representative images of IHC staining in tissues are shown. **E.** Kaplan-Meier analysis of the correlations between GRIM-19 expression and overall survival rate in GC patients. GRIM-19-positive: Strong plus moderate. Log-rank test was used for Kaplan-Meier survival analyses. Original magnification: ×100 (low power); ×400 (high power). ** *p*<0.01 and *** *p*<0.001 between the indicated two groups determined by paired student's *t* test.

To investigate the expression pattern of GRIM-19 in human GC, we first determined GRIM-19 transcript levels in 60 pairs of primary human GC tissues using qRT-PCR. We found that GRIM-19 mRNA levels were significantly decreased in GC tissues compared to paired adjacent tissues (Figure 1A). Next, we compared expression of GRIM-19 protein in larger GC cohorts to matched adjacent tissues (n=160) by immunohistochemistry (IHC) staining. IHC analysis showed that GRIM-19 protein was significantly depressed or lost in GC tissues compared to matched adjacent tissues (Figure 1B). GRIM-19 was broadly detected and exhibited strong staining in adjacent tissues, whereas it was negative or weakly positive in most GC tissues (Figure 1C), suggesting an aberrant gene silencing of GRIM-19 in GC. Moreover, in normal gastric mucosa and adjacent tissues, we observed a dominant cytoplasmic distribution of GRIM-19 in normal gastric epithelial cells, whereas in GC tissues, it was predominantly distributed in perinuclear regions or nuclei of tumor cells (Figure 1D), suggesting a different cellular distribution of GRIM-19 between GC tissues and non-tumor gastric tissues. In addition, we noted a relatively higher cancer cell intensity in GC tissues with lower GRIM-19 expression than that in normal gastric mucosa and adjacent tissues with higher GRIM-19 staining (Figure 1D), indicating that decreased GRIM-19 is associated with proliferative potential of GC cells.

### GRIM-19 is a potential prognostic biomarker of malignant progression of GC

To investigate the clinical significance of GRIM-19 expression in GC, we first analyzed the relationship between GRIM-19 protein expression and overall survival (OS) of GC patients. As shown in Figure 1E, Kaplan-Meier analysis revealed that GRIM-19 loss was significantly correlated with shorter OS. The 5-y OS probability was 70% in GRIM-19-positive patients, but only 30% (OS) in GRIM-19-negative patients. More importantly, higher GRIM-19 protein levels in tumor tissues could distinguish a subset of patients with increased risk of poor overall survival, demonstrating the clinical significance of GRIM-19 loss in the clinical outcomes of GC patients. Next, we investigated the correlation between GRIM-19 expression and clinicopathologic parameters in GC patients. Interestingly, GRIM-19 expression is independent of patients' gender, age, tumor differentiation and Lauren's histologic type, whereas lack of GRIM-19 significantly correlated with aggressive clinicopathologic features of GC patients including advanced clinical stage, lymph node metastasis, and *H. pylori* infection (Table 1), indicating that loss of GRIM-19 is associated with poor clinical outcomes of GC patients. Collectively, these data suggest that GRIM-19 is a potential prognostic biomarker of malignant progression in GC.

**Table 1:** Clinicopathologic characteristics and correlation with GRIM-19 expression

Variable	n (%)	GR19-Neg (%)	GR19-Pos (%)	*P value (Fisher's test)*
**Total Cases**	160	95 (59.4)	65 (40.6)	
**Gender**				
Male	97 (60.6)	55 (56.7)	42 (43.3)	0.415
Female	63 (39.4)	40 (63.5)	23 (36.5)	
**Age,y**				
≤58	72 (45.0)	48 (66.7)	24 (33.3)	0.107
>58	88 (55.0)	47 (53.4)	41(46.6)	
**Tumor size**				
≤5cm	86 (53.8)	51 (59.3)	35 (40.7)	0.395
>5cm	74 (46.2)	44 (59.5)	30 (40.5)	
**Differentiation**				
Well or Moderate	95 (59.4)	51 (53.7)	44 (46.3)	0.101
Poorly	65 (40.6)	44 (67.7)	21 (33.3)	
**Lauren's histologic type**				
Intestinal	93 (58.1)	57 (61.3)	36 (38.7)	0.626
Diffuse or Mixed	67 (41.9)	38 (56.7)	29 (43.3)	
**TNM stage**				
I-II	72 (45.0)	34 (47.2)	38 (50.8)	0.006^a^
III-IV	88 (55.0)	61 (69.3)	27 (30.7)	
**Lymph node metastasis**				
Absent	68 (42.5)	33 (48.5)	35 (51.5)	0.022^a^
Present	92 (57.5)	62 (67.4)	30 (32.6)	
**H.pylori infection**				
Positive	107 (66.9)	71 (66.4)	36 (33.6)	0.016^a^
Negative	53 (33.1)	24 (45.3)	29 (54.7)	

Note: The numbers in parentheses indicate the percentages of tumors with a specific clinical or pathologic feature for a given GRIM-19 subtype.

Abbreviation: GR19, GRIM-19; Neg, Negative; Pos, Positive.

a Statistically significant.

### GRIM-19 loss is an early molecular event in gastric carcinogenesis

**Figure 2:**
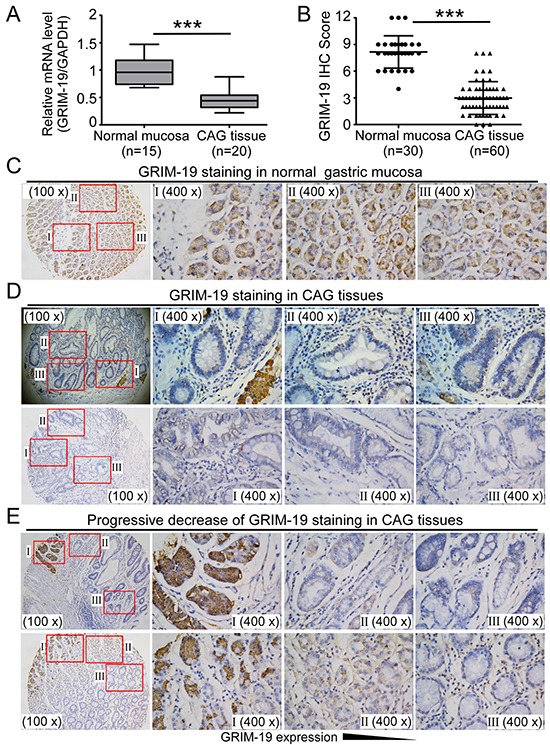
Decrease of GRIM-19 in human chronic atrophic gastritis (CAG) tissues **A.** Reduced GRIM-19 mRNA levels in CAG tissues. Total RNA was extracted from paraffin- embedded normal gastric mucosa (n=15) and CAG tissues (n=20). GRIM-19 mRNA was detected by qRT-PCR. GAPDH was used as an internal control. **B,C,D.** Decrease of GRIM-19 protein in CAG tissues. IHC staining was used to detect GRIM-19 protein expression and IHC scores for GRIM-19 were analyzed (**B**). Representative images of GRIM-19 staining in normal gastric mucosa tissues (**C**) and CAG tissues (**D**) are shown. **E.** Progressive decrease of GRIM-19 expression from normal gastric mucosa to intestinal metaplasia and dysplasia lesions of CAG tissues. Representative images of IHC staining in tissues are shown. Original magnification: 100× (low power); 400 ×(high power). * *p*<0.05, ** *p*<0.01 and *** *p*<0.001 between the indicated two groups determined by paired student's *t* test.

Chronic atrophic gastritis(CAG) appears to be the most consistent early lesion leading to GC [35]. To elucidate the role of GRIM-19 in the early stage of gastric carcinogenesis, we extended our work to investigate GRIM-19 expression in CAG tissues, a precursor of GC [35]. We found that GRIM-19 transcript and protein were markedly downregulated in CAG tissues compared to the normal gastric mucosa, as demonstrated by qRT-PCR analysis (Figure 2A) and IHC staining (Figure 2B). A strong GRIM-19 staining was detected in normal gastric mucosa tissues (Figure 2C), whereas very weak or no reactivity of GRIM-19 staining was observed in most CAG tissues, and an obvious reduction or absence of GRIM-19 expression was more frequent in intestinal metaplasia and dysplasia lesions of CAG tissues compared to the normal gastric mucosa (Figure 2D). Notably, a progressive decrease of GRIM-19 protein was observed from normal mucosa to mucosal atrophy, intestinal metaplasia and dysplasia lesions of CAG tissues (Figure 2E), suggesting a critical role of GRIM-19 in the homeostasis, maintenance and cell differentiation of gastric mucosa. In addition, we also found that downregulation of GRIM-19 is significantly correlated with *H. pylori* infection (Supplementary Table S1), strongly indicating that decrease of GRIM-19 is correlated with malignant transformation of CAG and is associated with *H. pylori* exposure. Collectively, these results suggest that GRIM-19 loss is an early molecular event in gastric carcinogenesis.

### GRIM-19 possesses tumor-suppressive function in human GC

**Figure 3:**
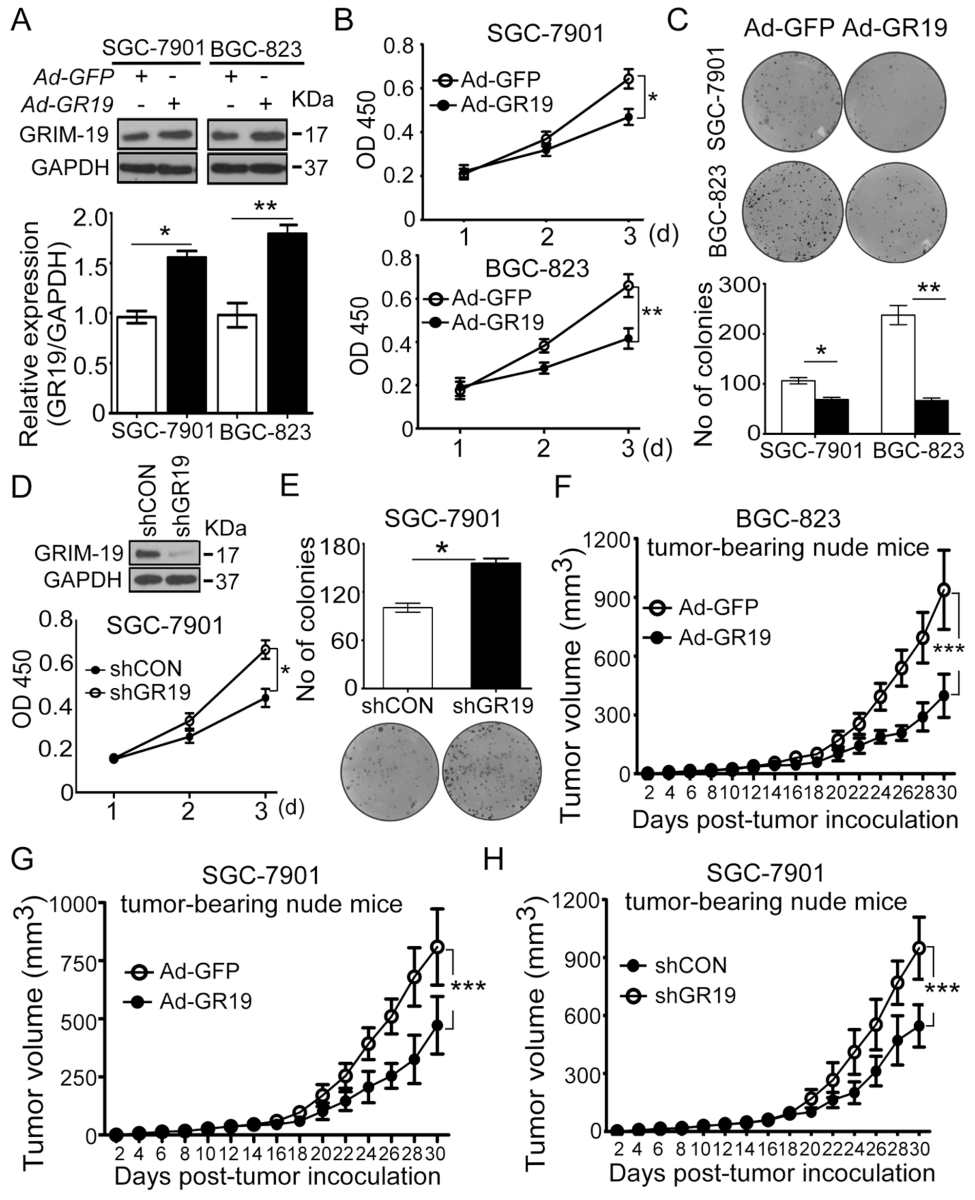
GRIM-19 possesses tumor-suppressive function in GC cells **A.** Ectopic GRIM-19 expression increased GRIM-19 protein levels in GC cell lines. Human GC cell lines were infected with adenovirus Ad-GR19 and Ad-GFP control. After 48h, GRIM-19 protein was confirmed by western blot. GAPDH was used as a loading control. **B,C.** Ectopic GRIM-19 expression inhibited cell proliferation and foci colony formation in GC cells. GC cells were transiently transfected with indicated adenovirus. After 24h, cell viability was detected by WST-1 assay at absorbance 450nm for 3 consecutive days (**B**) and colony formation ability (**C**) was determined by colony formation assay. Colony numbers were counted for 3 weeks after transfection. Representative images of colonies formed are shown. **D,E.** Deletion of GRIM-19 promoted cell proliferation and colony formation in GC cells. Endogenous GRIM-19 was stably deleted by shGR19 transfection in SGC-7901 cells. ShCON-transfected cells were used as control. GRIM-19 expression was confirmed by western blot and cell viability was detected by WST-1 assay at absorbance 450nm for 3 consecutive days (**D**). Colony formation ability was determined by colony formation assay (**E**). Colony numbers were counted after 3 weeks. Representative images of colonies formed are shown. **F,G.** Ectopic GRIM-19 expression inhibited GC tumor formation in nude mice. GC cells were transiently transfected with indicated adenovirus (Ad-GR19 & Ad-GFP). After 24 h, transfected cells (3 × 10^6^ /mice in 100μl PBS buffer) were subcutaneously injected into the rear flanks of the nu/nu mice (n=8 mice/group). Ad-GFP-infected cells were used as controls. Tumor size was measured every other day and presented as mean ± SD. **H.** Deletion of GRIM-19 expression promoted GC formation in nude mice. SGC-7901 shGR19 cells (1.5×10^6^ /mice in 100μl PBS buffer) were subcutaneously injected into the rear flanks of the nu/nu mice (n=8 mice/group). shCON-transfected cells were used as controls. Tumor size was measured every other day. Data are presented as mean ± SD. * *p*<0.05, ** *p*<0.01 and *** *p*<0.001 between the indicated two groups determined by paired student's *t* test or one-way analysis of variance.

To elucidate the functional role of GRIM-19 in the development and progression of GC, gain- and loss-of GRIM-19 function strategies were performed to analyze the role of GRIM-19 in GC cells. Re-expression of GRIM-19 was achieved by Adenovirus-GRIM-19 (Ad-GR19) in SGC-7901 and BGC-823 GC cell lines (Figure 3A), which have lower endogenous GRIM-19 expression compared to GES-1 cells and HEK-293 cells (Supplementary Figure S1). Ectopic expression of GRIM-19 significantly inhibited cell proliferation and colony formation in both cell lines (Figure 3B and 3C). To further test whether deletion of GRIM-19 could promote cell growth, a pool of specific GRIM-19 shRNA (shGR19) plasmid was used to stably delete endogenous GRIM-19 expression in SGC-7901 cells (Figure 2D, up). Knockdown of GRIM-19 dramatically promoted cell proliferation (Figure 2D, down) and colony formation in SGC-7901 cells (Figure 3E). Similarly, silencing of GRIM-19 dramatically increased cell proliferation and colony formation ability in GES-1 cells (Supplementary Figure S2).

In complementary *in vivo* xenograft mice models studies, ectopic GRIM-19 expression significantly inhibited tumor formation in SGC-7901 and BGC-823 cells (Figure 3F and 3G) whereas knockdown of GRIM-19 significantly promoted tumor growth in SGC-7901 cells, as shown in the xenograft tumor growth curve (Figure 3H). To verify the presence of GRIM-19 expression after subcutaneous injection in nude mice, GRIM-19 mRNA was confirmed by qRT-PCR in excised tumors tissues (Supplementary Figure S3A). These results strongly indicate that GRIM-19 possesses tumor-suppressive property in human GC.

### Cell cycle arrest and apoptosis are involved in tumor-suppressive functions of GRIM-19

**Figure 4:**
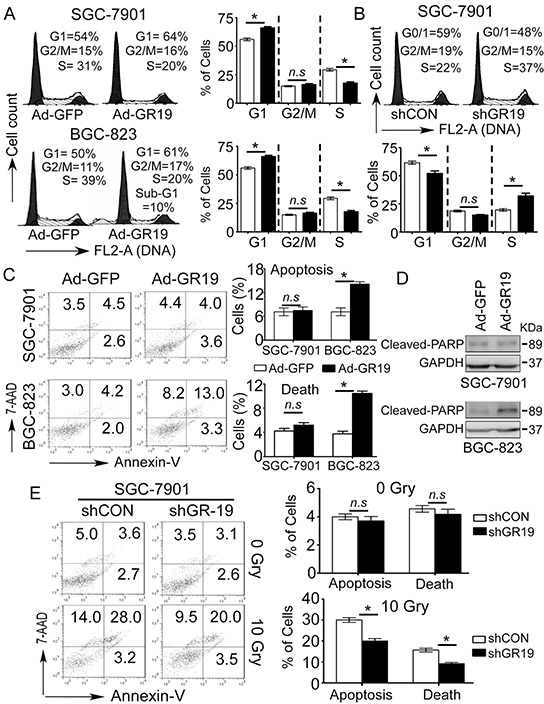
Cell cycle arrest and apoptosis are involved in tumor-suppressive function of GRIM-19 **A.** Ectopic GRIM-19 expression induced cell cycle G1/S phase arrest in GC cells. SGC-7901 and BGC-823 cells were transiently transfected with Ad-GR19 and Ad-GFP, respectively. After 72 h, cell cycle was examined by FACS analysis after RNAse treatment and PI staining. Representative images are shown. Data are presented as mean ± SD of 3 independent experiments. **B.** Knockdown of GRIM-19 promoted significant G1/S phase transition. SGC-7901 cells were stably transfected with shGR19 and shCON, respectively. Cell cycle was examined by FACS analysis after PI staining. Representative images are shown. **C,D.** Ectopic GRIM-19 expression induced cell type-dependent apoptosis in GC cells. GC cells were transiently transfected with Ad-GR19 and Ad-GFP respectively. After 72 h, cell apoptosis was measured using Annexin V-PE/7-AAD double staining (**C**) and apoptosis-relative cleaved-PARP was detected by western blot (**D**). Representative images are shown. **E.** Knockdown of GRIM-19 blunted sensitivity of GC cells response for irradiation treatment. ShGR19-transfected SGC-7901 cells were treated with or without 10 Gry irradiation for 10 min. After 48 h, cell apoptosis was measured using Annexin V-PE /7-AAD double staining. Representative images are shown. Data are presented as mean ± SD. *n.s*: no significance.* *p*<0.05, between the indicated two groups determined by paired student's *t* test. *n.s*: no significance.

To further determine whether cell cycle or cell apoptosis are involved in the suppressive effects of GRIM-19, we first evaluated the effect of GRIM-19 on cell cycle progression by flow cytometry. Ectopic expression of GRIM-19 significantly increased the fractions of cells in G1 phase and decreased the numbers in S phase in both SGC-7901 and BGC-823 cells (Figure 4A), whereas deletion of GRIM-19 in SGC-7901 cells promoted significant G1/S phase transition (Figure 4B). Furthermore, we also observed an apparent sub-G1 fraction in GRIM-19 overexpressing-BGC-823 cells (Figure 4A), indicating cell apoptosis or cell death may be involved in the tumor-suppressive functions of GRIM-19. Therefore, we next performed cell apoptosis analysis by Annexin-PE and 7-AAD double staining. Consistent with the results from cell cycle analysis, re-expression of GRIM-19 significantly induced both apoptotic and dead population in BGC-823 but not in SGC-7901 cells (Figure 4C), which was evidenced by the enhanced expression of cleavaged-PARP (Figure 4D) and Hoechst 33342 staining (Supplementary Figure S5). This contrast suggests that GRIM-19 induced-apoptosis is cell type-dependent.

To investigate whether GRIM-19 loss contributes to GC cell survival via resistance to apoptotic stimuli, we further assessed the response of GRIM-19 downregulation to X-ray irradiation in SGC-7901 cells. Interestingly, we found that deletion of GRIM-19 did not induce remarkable apoptosis, but significantly reduced the proportion of apoptotic and dead cells upon irradiation treatment in SGC-7901 cells (Figure 4E), suggesting that GRIM-19 loss inhibits GC cells from cell apoptosis response to apoptotic stimuli. These findings suggest that both cell cycle and apoptosis are involved in GRIM-19-mediated tumor-suppressive function in human GC.

### GRIM-19 suppresses STAT3 signaling by repressing STAT3 nuclear translocation

**Figure 5:**
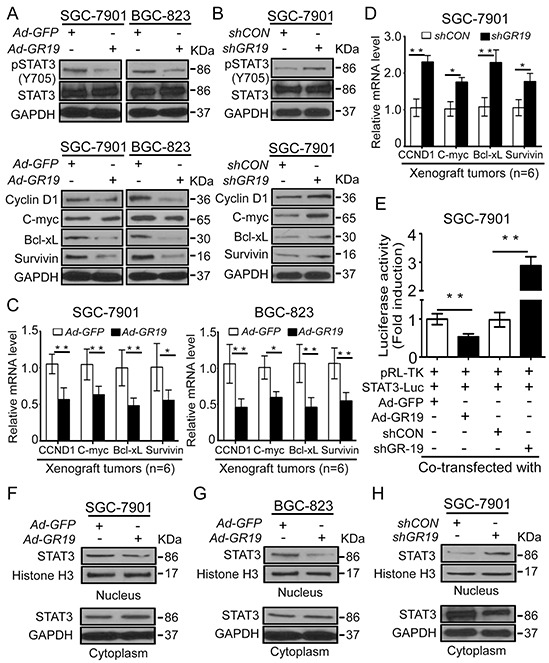
GRIM-19 suppresses STAT3 signaling via attenuating STAT3 nuclear translocation **A.** Ectopic expression of GRIM-19 decreased the expression of active STAT3 and its downstream targets in GC cells. SGC-7901 and BGC-823 were transiently transfected with Ad-GR19 and Ad-GFP for 72h, Phospho-STAT3(Try705), total STAT3 protein and its downstream targets including cyclin D1, c-Myc, Bcl-xL, and survivin were detected by Western blot. GAPDH was used as the loading control. **B.** Knockdown of GRIM-19 enhanced the expression of active STAT3 and its downstream targets in GC cells. SGC-7901 cells were stably transfected with shGR19 and shCON, respectively. Phospho-STAT3(Try705), total STAT3 protein and its downstream targets including Cyclin D1, c-Myc, Bcl-xL, and Survivin were detected by Western blot. GAPDH was used as the loading control. **C,D.** qRT-PCR for STAT3-responsive targets in xenograft tumor tissues. Xenograft tumors were established using indicated cells. STAT3-responsive genes were measured using qRT-PCR in xenograft tumors from GRIM-19-expressing GC cells (**C**) and GRIM-19-knockdown GC cells (**D**). GAPDH was used as internal control. Data are presented as mean ± SD. **E.** GRIM-19 repressed STAT3 transcriptional activation. GC cells were transiently transfected with the indicated reporter constructs along with shRNA plasmids or GRIM-19 expression vectors, respectively. pRL-TK *Reniila* plasmid was co-transfected to normalize transfection efficiency. At 24 h after transfection, the luciferase activity was quantified by dual luciferase assay. The data were presented as fold inductions of the ratio was normalized to *Renilla* luciferase activity. **F,G,H.** GRIM-19 inhibited STAT3 nuclear translocation. GC cells were transfected with indicated vectors. STAT3 expression was detected by Western blot from cytoplasmic and nuclear extracts in GRIM-19 expressing SGC-7901 cells (**F**), GRIM-19 expressing BGC-823 cells (**G**) and GRIM-19 knockdown SGC-7901 cells (**H**). GAPDH (for cytoplasmic protein) and Histone H3 (for nuclear protein) were used as loading control. *p<0.05, **p<0.01 between the indicated two groups determined by paired student's *t* test.

To understand the relationship between dysregulated GRIM-19 and aberrant STAT3 in GC cells, we first analyzed phospho-STAT3 (Try705) -pSTAT3 (Y705), an active form of STAT3, and its downstream targets including cyclin D1, Bcl-xL, C-myc and Survivin by Western blot. Ectopic GRIM-19 expression reduced pSTAT3 (Y705) levels and its downstream targets, but total STAT3 protein levels were not changed in both SGC-7901 and BGC-823 cells (Figure 5A). In contrast, knockdown of GRIM-19 markedly increased pSTAT3(Y705) levels and its downstream targets in SGC-7901 cells (Figure 5B). These target genes and GRIM-19 levels were further confirmed from corresponding xenograft tumor tissues by qRT-PCR (Figure 5C and 5D and Supplementary Figure S4A), which is consistent with the *in vitro* results. Next, STAT3 transcriptional activation was analyzed using luciferase reporter assay. We found that STAT3 transcriptional activities were remarkably inhibited by GRIM-19 overexpression, but were significantly enhanced by GRIM-19 knockdown in SGC-7901 cells (Figure 5E). Similar results were also observed in GES-1 and HEK-293 cells (Supplementary Figure S5). These results suggest that GRIM-19 inhibits active STAT3 and its downstream targets by suppressing STAT3 transcriptional activation.

To elucidate the mechanism behind GRIM-19's regulation of STAT3 activation, we analyzed the cellular distribution of STAT3 proteins in GRIM-19 re-expression and knock-down GC cells. Cellular fractions analysis revealed increased cytoplasmic STAT3 but decreased nuclear STAT3 in both GRIM-19 expressing SGC-7901 and BGC-823 cells (Figure 5F and 5G). Conversely, deletion of GRIM-19 dramatically increased nuclear STAT3 but decreased cytoplasmic STAT3 levels in SGC-7901 cells (Figure 5H). These results indicate that GRIM-19 suppresses STAT3 signaling by inhibiting STAT3 nuclear translocation in GC cells.

### Subcellular redistributions of GRIM-19 are associated with its inhibition to STAT3

**Figure 6:**
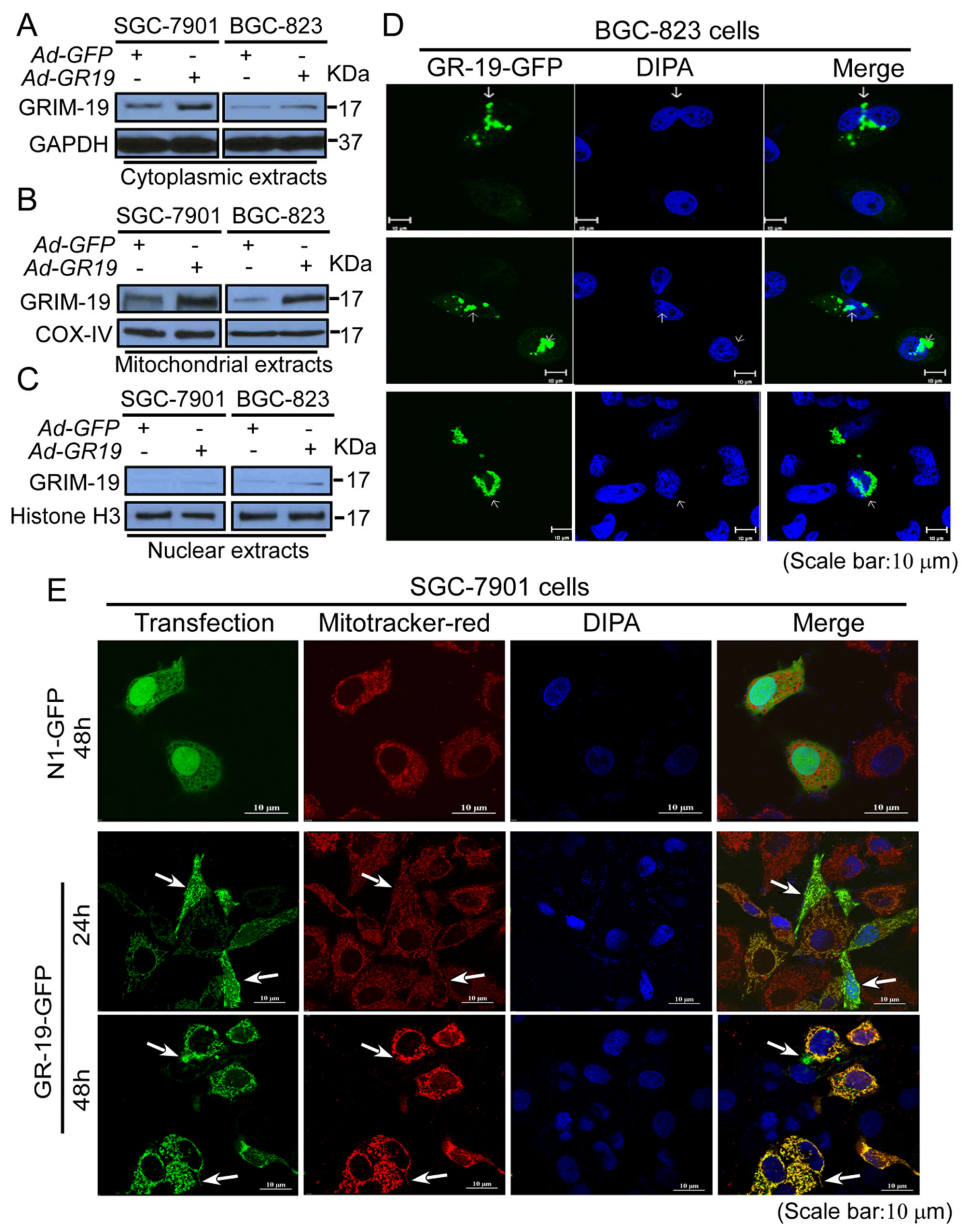
Subcellular redistribution of GRIM-19 is associated with its regulation to STAT3 nuclear distribution **A,B,C.** GRIM-19 expression enhanced GRIM-19 amounts in cytoplasm, mitochondria, and nucleus of GC cells. SGC-7901 and BGC-823 cells were transiently transfected with Ad-GR19 and Ad-GFP, respectively. After 72h transfection, Western blot was performed to detect GRIM-19 expression in cytoplasmic (**A**), mitochondrial (**B**), and nuclear extracts (**C**). GAPDH (for cytoplasmic protein), Histone H3 (for nuclear protein), and COX-IV (for mitochondrial protein) were used as loading control. **D.** Ectopic GRIM-19 expression induced clumps aggregates and nuclear invasion of GRIM-19. BGC-823 cells were transiently transfected with GRIM-19-GFP vector. The nuclei were counterstained by DAPI. GRIM-19-GFP (Green) was indicated by white arrow. **E.** Redistribution of GRIM-19 in the nucleus, perinuclear regions and non-mitochondrial cytoplasm of GC cells. SGC-7901 cells were transfected with GRIM-19-GFP or N1-GFP control vector. After indicated time point, mitochondria were labeled with Mitotracker-red probe (Red) and nucleus were counterstained with DAPI (Blue). Redistributed GRIM-19 (GFP) was indicated by white arrows. All images were captured with the laser-scanning microscope (Scale bar: 10μM). Representative images are shown.

To further explore how GRIM-19 regulates STAT3 nuclear translocation at the subcellular level, subcellular distribution of GRIM-19 was determined. Cellular fractions analysis showed that endogenous GRIM-19 is primarily distributed in both cytoplasmic and mitochondrial extracts, while a trace amount of GRIM-19 was detected in the nuclear extracts in both SGC-7901 and BGC-823 control cells (Figure 6A-5C). However, re-expression of GRIM-19 markedly increased GRIM-19 levels in cytoplasmic, mitochondrial, and nuclear extracts (Figure 6A–6C), indicating that re-expression of GRIM-19 induced its subcellular redistribution.

To determine whether the nuclear translocation or primary nuclear localization of GRIM-19 is responsible for its nucleus distribution, GRIM-19 expression was traced using GRIM-19-GFP plasmids, in which GRIM-19 was fused into the N-terminal of GFP protein. GRIM-19-GFP transfection resulted in densely clumped aggregates of GRIM-19 in BGC-823 cells, which co-localized with GRIM-19 staining (Supplementary Figure S6A). Importantly, we observed a typical nuclear invasion in GRIM-19-GFP transfected BGC-823 cells, resulting in an obvious morphological change of the nucleus (Figure 6D), In contrast, in SGC-7901 cells, GRIM-19-GFP presented a dense dot perinuclear distribution in the cytoplasm and partly in the nucleus, which was co-localized with enhanced GRIM-19 staining (Supplementary Figure S6B). These results suggest that nuclear invasion but not primary nuclear localization of GRIM-19 is responsible for its nuclear distribution.

To further define the exact cytoplasmic distribution of GRIM-19 in GC cells, cells were labeled with Mitotracker-red, a specific mitochondria probe. Post-transfection 24h, GRIM-19-GFP was found to be mainly co-localized with mitochondria, but also had a dot distribution in the nucleus (Figure 6E). However, at post-transfection 48h, GRIM-19-GFP triggered peri-nuclear clustering of mitochondria, resulting in an obvious morphological change of mitochondria. Notably, we further observed a visible non-mitochondria distribution in the cytoplasm of GRIM-19-GFP transfected cells (Figure 6E). These results suggest that GRIM-19 expression can induce subcellular redistribution in GC cells.

### STAT3 activation is critical to GRIM-19 loss-driven tumorigenesis in human GC

**Figure 7:**
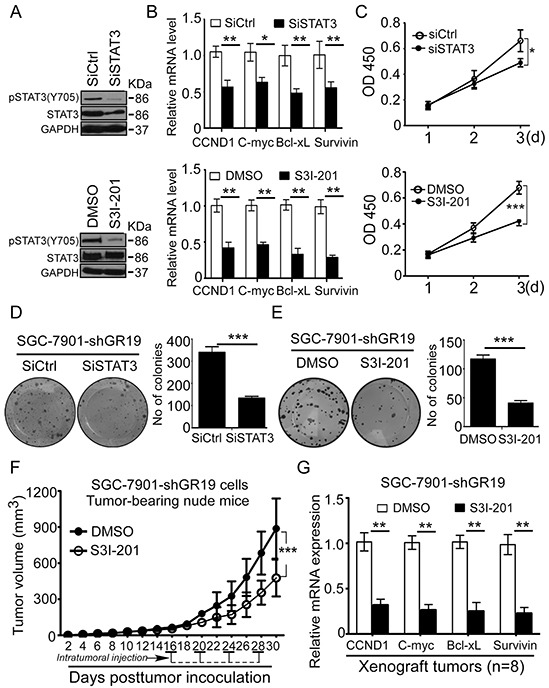
Blockage of STAT3 activation reverses GRIM-19 loss-driven tumor growth *in vitro* and *in vivo* **A,B.** Inhibition of STAT3 reduced GRIM-19 loss-induced STAT3 activation and STAT3-responsive genes expression. SGC-7901-shGR19 cells were treated with a pool of STAT3 siRNAs (siSTAT3) or STAT3 inhibitor S3I-201. Phospho-STAT3(Y705) and total STAT3 protein were detected by Western blot (**A**) and STAT3-responsive targets were analyzed with quantitative RT-PCR (**B**). GAPDH was used as an internal control. **C, D, E.** Abrogation of STAT3 activation reversed GRIM-19 loss-driven cell proliferation and colony formation *in vitro*. SGC-7901-shGR19 cells were treated with siSTAT3 or S3I-201. Cell viability was detected by WST-1 assay at absorbance 450nm for 3 consecutive days (**C**) and colony formation ability was determined by colony formation assay (**D, E**). Data are presented as mean ± SD of 3 independent experiments. Representative images of colonies formed are shown. **F, G.** Inhibition of STAT3 activation reversed GRIM-19 loss-derived tumorigenesis *in vivo*. Xenograft mouse models from SGC-7901-shGR19 were established as described in Materials & Methods. When tumor volumes had reached approximately 50 mm^3^, Mice (n=8 mice/group) were intratumorally injected four times with or without S3I-201, and tumor volume was calculated every two days (**F**). After indicated time, STAT3-responsive targets in excised xenograft tumors were analyzed using quantitative RT-PCR (**G**). Data are presented as mean ± SD. * *p*<0.05, ** *p*<0.01 and *** *p*<0.001 between the indicated two groups determined by paired student's *t* test.

To determine whether STAT3 is required for the GRIM-19 loss-driven tumorigenesis, we tested whether inhibition of STAT3 activation could attenuate or eliminate GRIM-19 loss-induced GC growth. We used pooled STAT3 siRNAs (siSTAT3) or STAT3 inhibitor- S3I-201 to inhibit STAT3 activation (Figure 7A) and its downstream gene expression (Figure 7B) in GRIM-19 knockdown-SGC-7901 (SGC-7901-shGR19) cells. Both STAT3 siRNA and S3I-201 treatment significantly abrogated GRIM-19 loss-induced cell proliferation (Figure 7C) and colony formation (Figure 7D and 7E). To further assess the role of STAT3 *in vivo*, nude mice bearing SGC-7901-shGR19 tumors (approximate 50 mm^3^) were intratumorally injected with or without S3I-201. As expected, GRIM-19 knockdown-driven tumorigenesis was markedly attenuated upon intratumoral treatment of S3I-201 *in vivo* (Figure 7F). Quantitative RT-PCR analysis on the xenograft tumor tissues showed that STAT3-responsive targets were significantly decreased after S3I-201 treatment (Figure 7G), whereas GRIM-19 expression was not affected (Supplementary Figure S3B). These data suggest that STAT3 is required for GRIM-19 loss-driven tumorigenesis.

## DISCUSSION

Elucidating the factors causing aberrant STAT3 activation in GC has been considered as a key to revealing critical mechanisms of gastric carcinogenesis. To date, three biological STAT3 inhibitors including suppressor of cytokine signaling 3 (SOCS3) [39, 40, 42], protein inhibitor of activated STAT3 (PIAS3) [41, 42], and GRIM-19 [26–29, 42] have been reported. However, tumor-suppressive roles of SOCS3 and PIAS3 have not yet been established [42]. It is therefore of interest in our current study to focus on the role of GRIM-19 in gastric carcinogenesis and its correlation with STAT3 activation in GC cells. In the current study, we investigated clinical implications of GRIM-19 expression in the pathogenesis of GC and precancerous chronic atrophic gastritis (CAG). Our results suggested that GRIM-19 loss is both involved in the aggressive tumorigenesis of GC and malignant transformation of CAG pathology. In primary human GC tissues, we observed a frequent decrease or loss of GRIM-19 expression which is associated with aggressive clinicopathologic features of patients with GC, suggesting that GRIM-19 may serve as a useful prognosis biomarker in the progression of GC. Importantly, besides its decrease in GC, GRIM-19 expression was also decreased in CAG tissues with progressive decrease in parallel with malignant transformation of CAG, suggesting that GRIM-19 inactivation, as an earlier molecular event in gastric carcinogenesis, may be involved in the homeostasis, maintenance or cell differentiation of gastric mucosa epithelial cells. These studies collectively suggest that GRIM-19 is an valuable prognostic biomarkers for predicting the outcomes of human GC as well as for malignant transformation of CAG. However, at this stage, it remains unclear what contributes to GRIM-19 loss in GC and CAG tissues. Recent studies have reported that gene mutation or promoter hypermethylation may be involved in the inactivation of GRIM-19 [30–34, 36]. Our preliminary data showed that tumor-derived GRIM-19 mRNA seems to harbor no mutations, and no significant deletions in the GRIM-19 gene were observed in tumor and normal genomic DNA (data not shown), suggesting that gene mutations may not be involved in GRIM-19 inactivation. Interestingly, we noted that both mRNA and protein levels of GRIM-19 were markedly decreased in GC and CAG tissues, which are associated with *H. pylori* infection. Therefore, GRIM-19 loss may be due to exposure to *H. pylori*, which has been shown to increase DNA methyltransferase activity and promote DNA methylation [37, 38]. However, the direct link between GRIM-19 loss and DNA methylation needs to be investigated in future studies.

Identifying functional role of new biomarkers involved in aberrant STAT3 activation in GC may shed light on potential therapeutic targets for GC. In this study, using gain- and loss-of-function strategies, we demonstrated an oncogenic role of GRIM-19 loss in promoting GC tumorigenesis partly by activating a STAT3-dependent pathway. Our study showed that GRIM-19 functions as a novel tumor suppressor by inducing cell cycle arrest and apoptosis in tumorigenesis of GC. Considering that the inhibitory effects of GRIM-19 on STAT3 activation may be tumor or cell type-dependent [20], we investigated whether disturbed STAT3 is involved in GRIM-19 loss-driven GC tumorigenesis in GC. We first demonstrated that GRIM-19 inhibited STAT3 activation and its downstream targets *in vitro* and *in vivo* and revealed that STAT3 nuclear translocation contributes to GRIM-19-mediated suppression of STAT3 transcriptional activation. Moreover, we confirmed that STAT3 activation is required for GRIM-19 loss-driven tumorigenesis of GC cells through pharmacological STAT3 intervention. However, pharmacological inhibition of STAT3 did not completely abrogate GRIM-19 loss-driven tumorigenesis, suggesting that the full capacity of GRIM-19 loss to tumor growth may extend beyond its ability to activate STAT3. Since GRIM-19 loss also contributed to the switch between oxidative and glycolytic pathways [36, 42, 43], while aberrant glycolytic metabolism provides tumor growth advantages [44–46]. Therefore, compensatory activation of these contributions by GRIM-19 loss may provide a reasonable account for the GRIM-19 loss-driven tumorigenesis.

In addition, our current studies also revealed unrecovered details regarding subcellular redistributions of GRIM-19 in GC cells, which could provide a reasonable explanation for the suppression of GRIM-19 to STAT3 nuclear distribution. Recently, the physical interaction between GRIM-19 and STAT3 and their intracellular co-localization has been well clarified [16]. However, the cellular localization of GRIM-19, which is critical for the inhibition of GRIM-19 to STAT3 activation, is still controversial [20, 21, 27]. In this study, we revealed an opposite nuclear distribution between GRIM-19 and STAT3 after GRIM-19 re-expression by Western blot. Notably, we observed subcellular redistributions of GRIM-19 characterized by nuclear invasion, non-mitochondrial cytoplasmic distribution as well as perinuclear aggregates in GRIM-19-expressing GC cells. Based on these results, therefore, there are the most two possibilities underlying the inhibition of GRIM-19 to STAT3. In the cytoplasm, non-mitochondrial GRIM-19 is preventing STAT3 from entering nucleus by binding cytoplasmic STAT3; In the nucleus, invasive GRIM-19 blocks STAT3 activation by direct binding nuclear STAT3, ultimately inhibiting STAT3 pathway. These findings would facilitate better understanding of the inhibitory details of GRIM-19 to STAT3 activation, further strengthening the hypothesis that GRIM-19 is a STAT3 inhibitor in human GC.

In summary, our studies suggest that mitochondrial GRIM-19 could not only serve as a new prognostic biomarker, but also as a potential therapeutic target for STAT3-dependent carcinogenesis of GC. An ongoing study in our lab is investigating the complex mechanisms of GRIM-19 in initiation and progression of both GC and CAG using a gastric-specific GRIM-19 knockout mice model, which would provide a more comprehensive understanding of its role in GC pathogenesis.

## MATERIALS AND METHODS

### Patients and tissue samples

Human GC and matched non-tumorous tissues (n=160), chronic atrophic gastritis (CAG) tissues (n=60), and normal gastric mucosal specimens (n=30) were obtained from Dept of pathology, Xinqiao hospital of Third Military Medical University from 1998 to 2009. No patients had received any preoperative chemotherapy or irradiation before the operation. Histological features of these specimens were confirmed by clinical pathologists, and *H. pylori* infection in tissue samples was available. The GC patients had well-documented clinical histories and follow-up information. All specimens were handled anonymously according to ethical standards. The study protocol was approved by the Ethical Review Committee of the Chongqing Medical University. Details of patient characteristics are provided in Supplementary Table S1. All tissues were incorporated into tissue micro-arrays (1.0-mm diameter and 5um per spot), and GRIM-19 expression was detected using IHC staining (Details see Supplementary Materials & Methods).

### Cell lines and reagents

The immortalized normal gastric epithelial cell line GES-1, human embryonic kidney HEK-293 cells, human GC SGC-7901 and BGC-823 cell lines were obtained from the Cell Bank of the Chinese Academy of Science (Shanghai, China) and the American Type Culture Collection (ATCC, Manassas, VA, USA). Cells were cultured in DMEM or RPMI 1640 (Gibco BRL, Rockville, MD, USA) supplemented with 10% fetal bovine serum, 100 U/ml penicillin and 100 μg/ml streptomycin at 37°C in a humidified chamber containing 5% CO_2_. The cells were tested regularly for mycoplasma (New MycoProbe Mycoplasma Detection Kit, R&D Systems). All chemical reagents were purchased from Sigma-Aldrich (St. Louis, MO, USA) unless otherwise indicated.

### Recombinant adenovirus, recombinant plasmids, small interfering RNA and transfection

Generation and transfection of recombinant adenovirus-GRIM-19 (Ad-GR19) and GFP control (Ad-GFP) were performed as described previously [21]. A pooled GRIM-19 shRNA (shGR19) plasmids (Santa Cruz Biotechnology, Santa Cruz, CA) was used to knockdown GRIM-19 expression in indicated cells and a scramble shRNA (shCON) was used as control. ShRNA plasmids were transfected into indicated cells and resistant colonies were selected with puromycin (1ug/ml). Individual clones were isolated and expanded in the selection medium. Pooled STAT3 siRNA (siSTAT3) and Control siRNA (siCtrl) were purchased from Santa Cruz Biotechnology. STAT3 luciferase reporter plasmid pSTAT3-TA-luc was purchased from Beyotime Inc (Haimen, China). GRIM-19-GFP recombinant was generated by inserting GRIM-19 cDNA to the GFP upstream of pEGFP-N1 plasmids (Clontech) [21]. Transfection of plasmids or siRNAs was performed using FuGENE HD transfection reagent (Roche, Basel, Switzerland) according to the manufacturer's instructions.

### Cell viability assay and colony formation assay

Cells seeded on a 12-well plate with 50% to 80% confluence were transiently transfected with indicated vectors. After 24 hours, the cells were subjected to cell viability assay and colony formation assay as previously described [21, 47] (Details see Supplementary Materials & Methods).

### Cell cycle, apoptosis analysis and irradiation

Cell cycle and cell apoptosis analysis was performed by flow cytometry (FACSCalibur, BD) as previously described [21, 47]. Cells in a 6-well plate were transfected with indicated vectors for 48 hours and subject to cell cycle analysis. In some case, irradiation was carried out with a RS2000 biological X-Ray irradiator (Rad Source Technologies Inc, USA) producing 160 kVp X-rays on a 6 well plate according to the manufacturer's instructions. Cells undergoing apoptosis were identified using the Annexin V-PE /7-AAD apoptosis Kit (Invitrogen, Carlsbad, CA, USA) according to the manufacturer's instructions, and the data were analyzed with FlowJo software (Tree Star, Ashland, OR).

### RNA extraction and quantitative RT-PCR

RNA from formalin-fixed, paraffin-embedded tissues was extracted using RNeasy FFPE Kit (QIAGEN, Hilden, Germany) according to the manufacturer's instructions. RNA extraction from cells and xenograft tissues and quantitative RT-PCR were performed as previously described [21, 47] (Details see Supplementary Materials & Methods). The sequences of primers used are listed in Supplementary Table S2.

### Isolation of mitochondria and cell fractions, protein extracts, and western blot

Cell mitochondria were isolated using mitochondria isolation kit (Pierce, Rockford, IL, USA) according to the manufacturer's instructions. Total cell lysates and mitochondrial proteins were extracted using protease inhibitor cocktail-containing RIPA lysis buffer (Roche). Cell fractions were prepared using NE-PER Nuclear and Cytoplasmic Extraction Reagents (Pierce). Protein concentration was determined by the BCA Protein assay kit (Pierce). Western blot was performed to detect indicated proteins as previously described [21, 47]. All antibodies used are listed in Supplementary Table S3.

### Luciferase reporter assay

Cells were seeded in 24-well plates at a concentration of 1 ×10^5^ cells per well and were transfected with the pSTAT3-TA-Luc, shRNA plasmids or gene expression vectors. pRL-TK *Reniila* plasmid (10 ng) was co-transfected to normalize transfection efficiency. The luciferase activity in the cells was quantified using a dual luciferase assay system (Promega, Madison,WI, USA) 24 hours after transfection. The data were presented as fold inductions of the ratio was normalized to *Renilla* luciferase activity.

### Immunofluorescence

Immunofluorescence analyses were performed as previously described [21] (Details see Supplementary Materials & Methods).

### Xenograft mouse models

BALB/C nude (nu/nu) mice (6-8 weeks, Female, SPF degree, 20 ± 3 g) were purchased from Laboratory Animal Center of Chongqing medical University (Chongqing, China). All procedures were approved by the Institutional Animal Care Committee. Xenograft mouse models were established by subcutaneous (s.c.) injection (Details see Supplementary Materials & Methods).

### Statistics

Statistical calculations were carried out with the GraphPad Prism 5 (GraphPad Software). All data were expressed as means ± SD. The Fisher's exact test was used to analyze the correlation between GRIM-19 expression and clinicopathologic features. Survival curves were generated according to the Kaplan–Meier method and the statistical analysis was done by log-rank test. Two-paired Student *t* test was used to analyze data from cell growth, foci formation, and tumor formation in nude mice and gene expression analysis. The value of *p*<0.05 was considered statistically significant.

## SUPPLEMENTARY FIGURES AND TABLES


