# Supplementary material for: Mitochondrial GRIM-19 as a potential therapeutic target for STAT3-dependent carcinogenesis of gastric cancer
Source: Oncotarget. 2016 May 4;7(27):41404–20. doi: 10.18632/oncotarget.9167 (PMC5173068; doi:10.18632/oncotarget.9167)
Supplement: Supplementary file 1 [file oncotarget-07-41404-s001.pdf]

SUPPLEMENTARY FIGURES AND TABLES

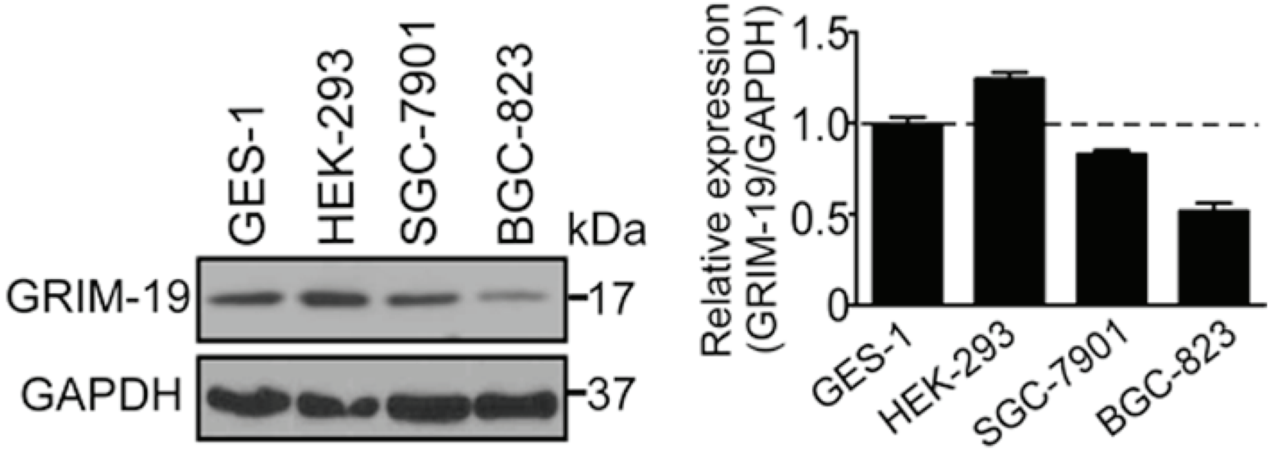

Supplementary Figure S1: GRIM-19 expression in human GC cell lines. GRIM-19 expression was tested using Western blot in human GC SGC-7901 and BGC-823 cell lines. Human gastric mucosal epithelial cell line GES-1 and human embryonic kidney HEK-293 cells serves as control cells, and GAPDH as the internal control. Representative images are shown. Data are presented as mean ± SD of 3 independent experiments.

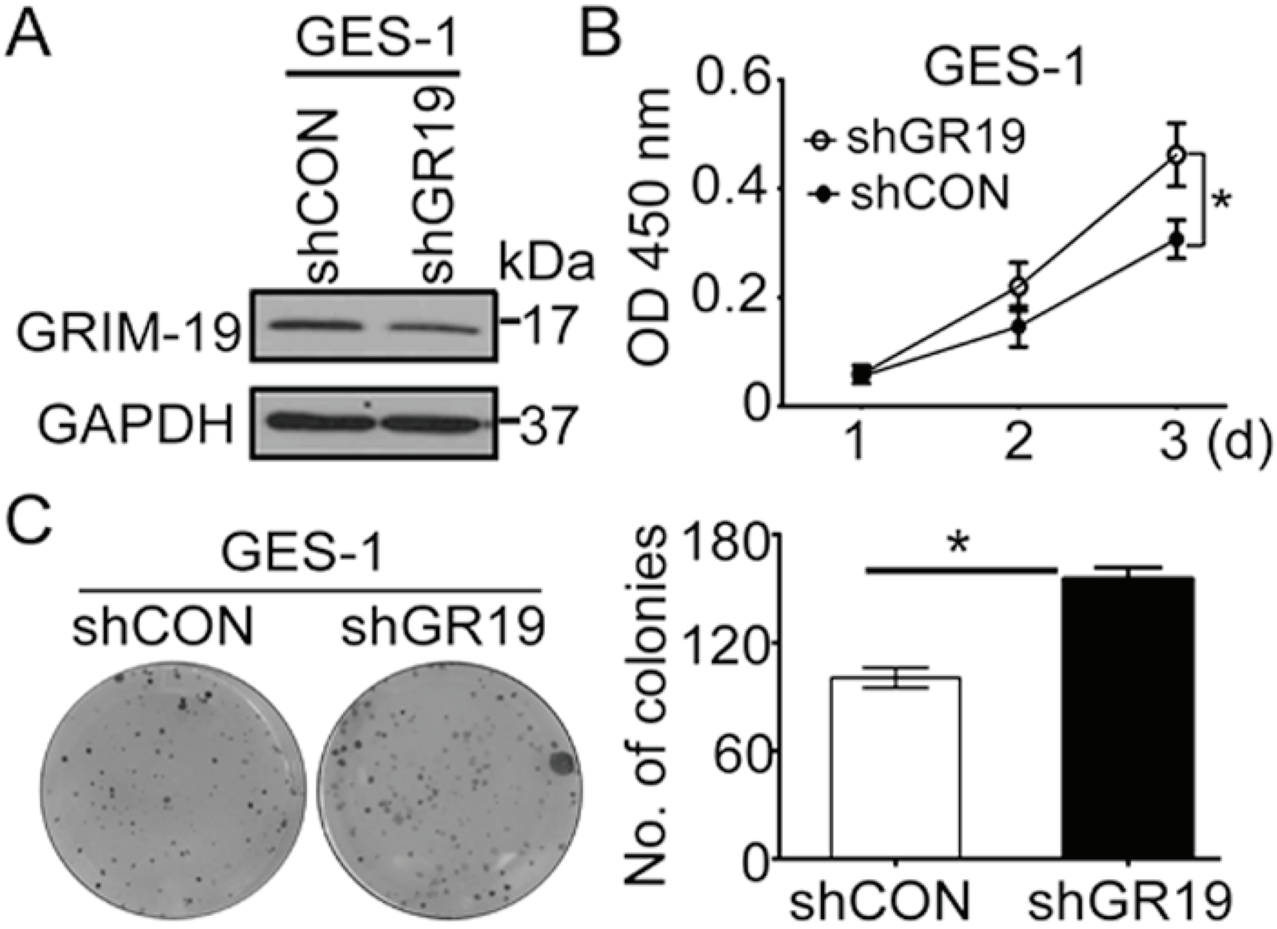

Supplementary Figure S2: Abrogation of GRIM-19 enhances cell proliferation and colony formation in GES-1 cells. GES-1 cells were transiently transfected with shGR19 and shCON vectors, respectively. At 72 h after tranfection, GRIM-19 expression was detected by Western blot **A.** GAPDH was used as an internal control. Cell viability was determined by WST-1 assay for 3 consecutive days **B.** and colony formation assay was used to examine colony formation ability **C.** Representative images of colonies formed are shown. * $p<0.05$ between the indicated two groups determined by paired student's *t* test. Data are presented as mean ± SD of 3 independent experiments.

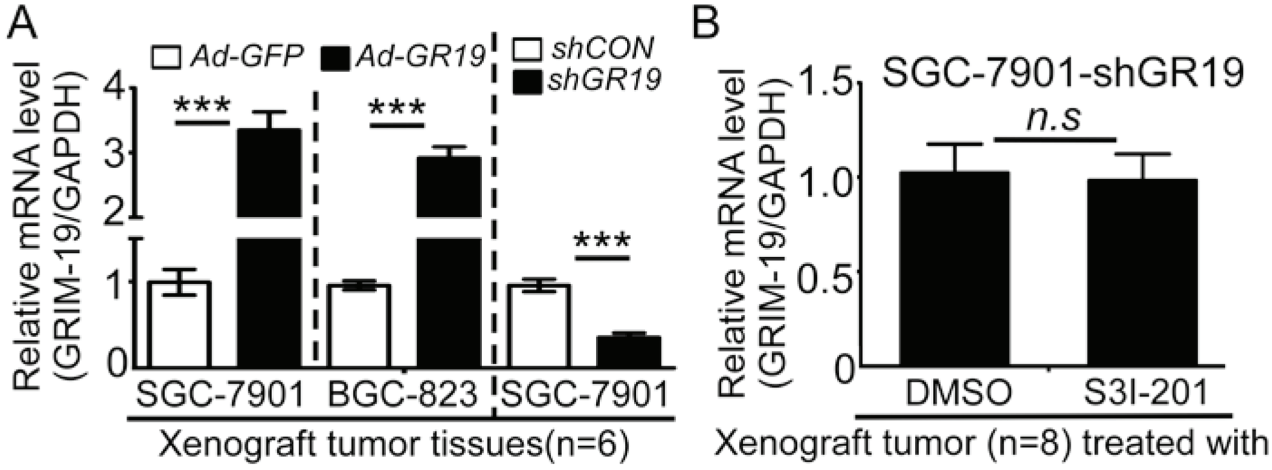

Supplementary Figure S3: Quantitative RT-PCR for GRIM-19 in xenograft tumor tissues. GRIM-19 mRNA was measured by qRT-PCR from xenograft tumor tissues established using indicated GC cells **A.** and treated with S3I-201 **B.** GAPDH was used as an internal control. *** $p<0.001$ between the indicated two groups determined by paired student's *t* test. Data are presented as mean ± SD. *n.s*: no significance.

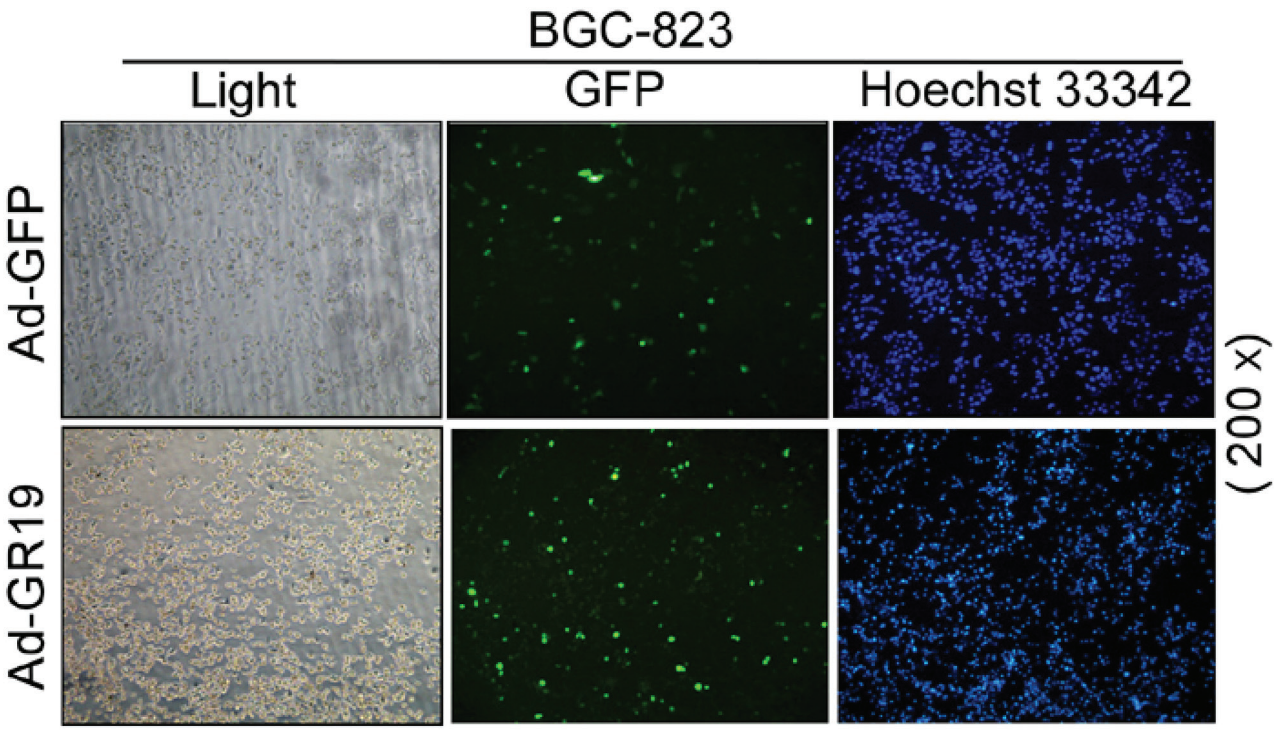

Supplementary Figure S4: Hoechst 33342 staining for Ad-GR19-transducted GC cells. BGC-823 cells were transiently transducted with Ad-GFP and Ad-GR19 (Green), respectively. Post-tranfection 48h, nucleus were counterstained with Hoechst 33342 (Blue) and images were captured with an inverted fluorescent microscope (200× magnification). Representative images are shown.

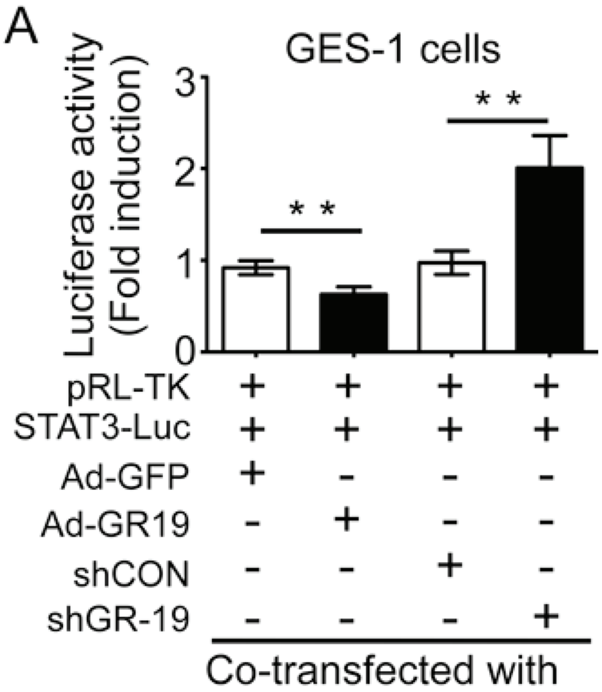

Supplementary Figure S5: GRIM-19 represses STAT3 transcriptional activation in GES-1 and HEK-293 cells. STAT3 reporter constructs were transiently transfected into GES-1 **A.** and HEK-293 cells **B.** along with GRIM-19 shRNA plasmids or Ad-GRIM-19 expression vectors, respectively. pRL-TK *Reniila* plasmid was co-transfected to normalize transfection efficiency. After 24 h transfection, the luciferase activity was quantified by dual luciferase assay. The data were presented as fold inductions of the ratio was normalized to *Renilla* luciferase activity. ** p<0.01 between the indicated two groups determined by paired student's t test. Data are presented as mean ± SD of 3 independent experiments.

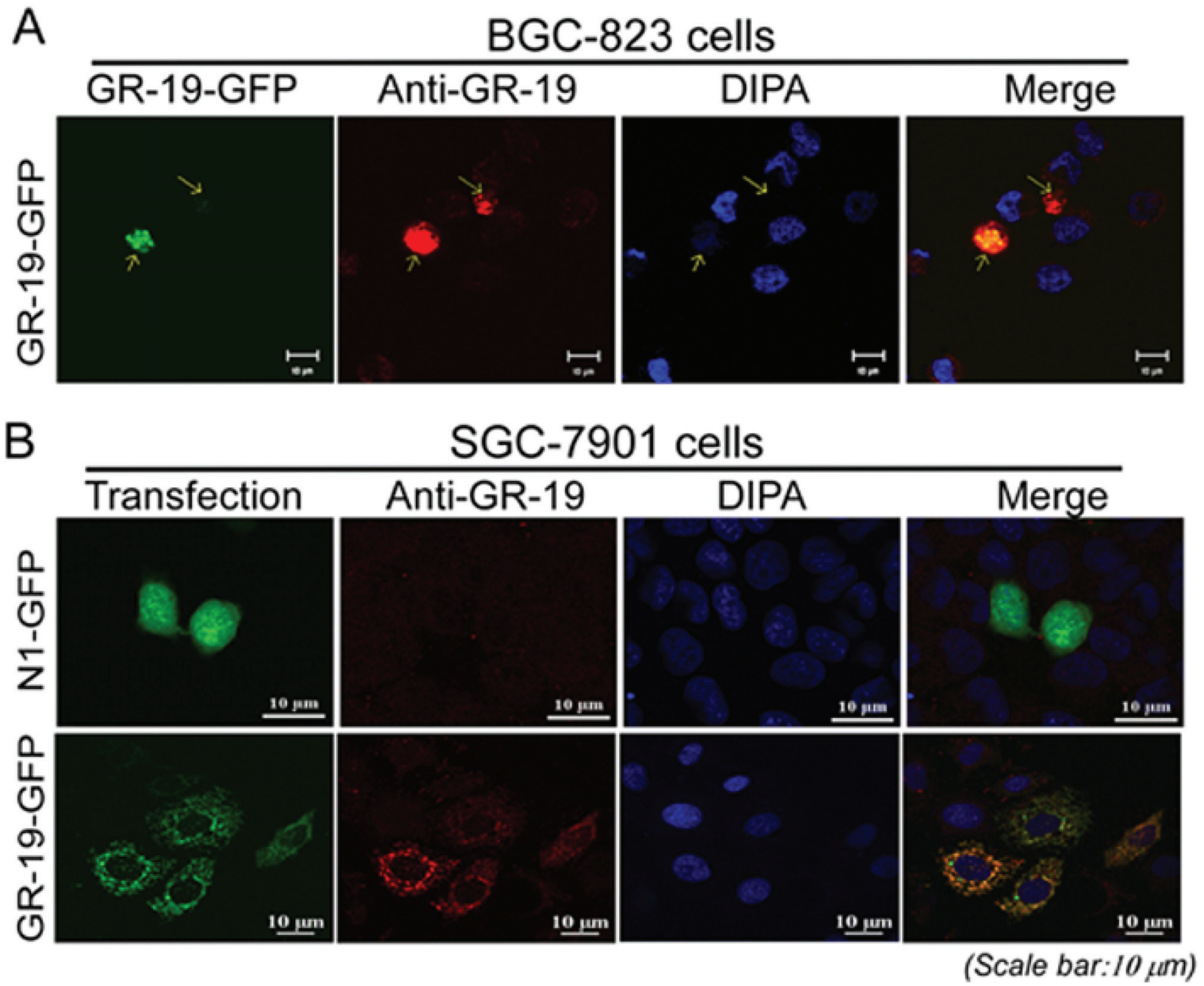

Supplementary Figure S6: Immunofluorescence for GRIM-19 in GRIM-19-GFP transfected GC cells. BGC-823 cells were transiently transfected with GRIM-19-GFP expressing vector **A.** SGC-7901 cells were transiently transfected with GRIM-19-GFP and N1-GFP control vectors **B.** respectively. GRIM-19 expression was detected by indirect immunofluorescence staining with the GRIM-19-specific antibody (Red). Nucleus were counterstained with DIPA (Blue) and images were captured by the laser scanning microscope (Scale bar:10μM). Representative images are shown.

Supplementary Table S1: Association of GRIM-19 expression with *H.pylori* infection of human CAG patients

Variable	n (%)	GR19-Neg (%)	GR19-Pos (%)	P value *(Fisher's test)*
**Total Cases**	60	41 (68.3)	19 (31.4)	
***H.pylori* infection**				
Positive	39 (65.0)	31 (75.6)	8 (24.4)	0.019[a]
Negative	21 (35.0)	10 (47.6)	11 (52.4)	

Note: [a] Statistically significant.

Supplementary Table S2: Primers used for Quantitative RT-PCR in this study

Gene (Genbank Accession number)	Primer sequences (5'-3')	Size (bp)
GRIM-19 (NM_015965.6)	Forward: ACCGGAAGTGTGGGATACTG; Reverse: GCTCACGGTTCCACTTCATT;	194
CCND1 (NM_053056.2)	Forward: GATGCCAACCTCCTCAACGAC; Reverse: CTCCTCGCACTTCTGTTCCTC;	171
BIRC5 (Survivin) (NM_001168.2)	Forward: AGAACTGGCCCTTCTTGGAGG; Reverse: CTTTTTATGTTCCTCTATGGGGTC;	170
Bcl-xl (NM_001033670)	Forward: GGTCGCATTGTGGCCTTCTT; Reverse: CTCTCGGCTGCTGCATTGTT;	196
C-myc (NM_002467.4)	Forward: TGCCACGTCTCCACACATCA; Reverse: CCTGGGGCTGGTGCATTTT;	124
GAPDH (NM_002046.5)	Forward: AGCCACATCGCTCAGACAC; Reverse: GCCCAATACGACCAAATCC;	66

Supplementary Table S3: Antibodies used for western blot in this study

Antibodies	Catalog number (Dilution ratio)	Size (kDa)
GRIM-19	Santa Cruz (Sc-47027) dilution 1:600	17
Cleaved-PARP	Cell signling(# 5625) dilution 1:1000	89
Cyclin D1	Cell signling(# 2978) dilution 1:1000	36
Survivin	Cell signling(# 2808) dilution 1:1000	16
Bcl-xl	Cell signling(# 2764) dilution 1:1000	30
C-myc	Cell signling(# 13987) dilution 1:1000	65
STAT3	Cell signling(# 8768) dilution 1:1000	86
STAT3 (Try705)	Cell signling(# 9145) dilution 1:1000	86
COX-IV	Cell signling(# 4844) dilution 1:1000	17
Histone H3	Cell signling(# 4499) dilution 1:1000	17
GAPDH	Cell signling(# 5174) dilution 1:1000	37
